# Three-dimensional variations of the slab geometry correlate with earthquake distributions at the Cascadia subduction system

**DOI:** 10.1038/s41467-018-03655-5

**Published:** 2018-03-23

**Authors:** Haiying Gao

**Affiliations:** Department of Geosciences, University of Massachusetts Amherst, 627 North Pleasant St, Amherst, MA 01003 USA

## Abstract

Significant along-strike variations of seismicity are observed at subduction zones, which are strongly influenced by physical properties of the plate interface and rheology of the crust and mantle lithosphere. However, the role of the oceanic side of the plate boundary on seismicity is poorly understood due to the lack of offshore instrumentations. Here tomographic results of the Cascadia subduction system, resolved with full-wave ambient noise simulation and inversion by integrating dense offshore and onshore seismic datasets, show significant variations of the oceanic lithosphere along strike and down dip from spreading centers to subduction. In central Cascadia, where seismicity is sparse, the slab is imaged as a large-scale low-velocity feature near the trench, which is attributed to a highly hydrated and strained oceanic lithosphere underlain by a layer of melts or fluids. The strong correlation suggests that the properties of the incoming oceanic plate play a significant role on seismicity.

## Abstract (web summary)

Variations in seismicity are observed at subduction zones, but the oceanic sides remain poorly resolved. Here, the author presents tomographic results of the Cascadia subduction system demonstrating that there are significant variations of the oceanic lithosphere along the subduction zone.

## Introduction

**Fig. 1:**
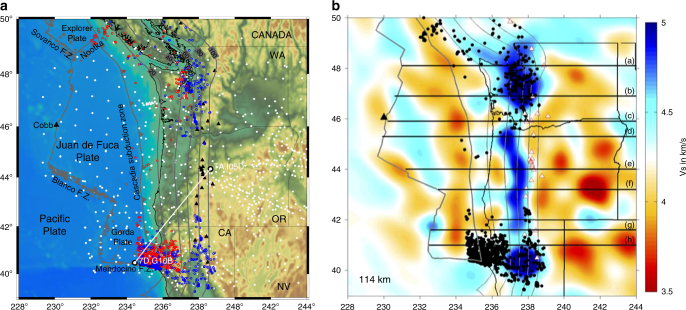
Correlation of the seismicity in Cascadia with the geometry of the subducting slab. **a** Distribution of seismic stations (white dots) used for full-wave ambient noise tomography and regional earthquakes within the study region. The gray stars mark the offshore earthquakes with magnitude greater than 4.5 from 2000 to 2017 from the Global CMT catalog. The red and blue circles correspond to the *M* ≥ 3.0 earthquakes from 1975 to 2005 below and above the plate interface, respectively^11^. The black triangles mark the Cascade arc volcanoes and the Cobb axial seamount in the central Juan de Fuca ridge. The gray contours are the plate interface from 20 to 100 km depth^10^. The station pair 7D.G10B−TA.I05D is used in Fig. 2. **b** Shear-wave velocity model imaged in this study at 114 km depth. The black dots are the background seismicity in Cascadia. The black lines mark the profile locations used in Fig. 4

The relatively young, warm, and thin oceanic Juan de Fuca and Gorda plates are subducting beneath the North American continent along the Cascadia subduction zone (Fig. 1), representing an endmember of the subduction systems. Shallow dehydration is expected within the Juan de Fuca and Gorda subducting slabs^1, 2^, resulting in fewer megathrust earthquakes in Cascadia, with the most recent one occurring in A.D. 1700^3, 4^. The lack of instrumentally recorded megathrust earthquakes in Cascadia also indicates that the seismogenic zone is currently fully locked^5^. Previous studies at global subduction zones^5–8^ have suggested that along-strike variations of earthquake distributions may indicate different degrees of slab dehydration, thermal structures of the incoming plate, coupling of the plate interface, curvature of the subducting slab, the subduction parameters, as well as subducting features (e.g., seamounts, fracture zones, and sediments). For example, in the Alaska subduction zone more subduction-related earthquakes are detected where hydration of oceanic mantle due to the bending-related faultings is observed^9^. Along the South American subduction zone, structural variations within the subducting and overriding plates significantly contribute to the heterogeneity of the subduction earthquakes along strike^6^.

In the Cascadia subduction zone, the background seismicity can be divided into two groups, the continental crustal earthquakes and the intraslab earthquakes, in terms of their locations relative to the plate interface^10, 11^ (see Fig. 1). In general, the seismicity is clustered in northern and southern Cascadia and is lacking in the central part, demonstrating remarkable variation patterns along strike and with depth. More specifically, abundant intraslab and continental earthquakes are observed in northwestern California at depths shallower than 40 km, widely spreading from the trench landward to the arc region. Beneath western Washington, the intraslab earthquakes extend from 20 km depth down dip to 80 km depth, and continental earthquakes mainly occur above the 40–100 km slab depths. Beneath western Oregon, there is a lack of both types of earthquakes, with an exceptional cluster of small earthquakes off central Oregon^12, 13^ (Fig. 1).

A few factors have been attributed to explain the observed along-strike variation patterns of seismicity at Cascadia. The relatively shallow and widespread seismicity in northern California was proposed to reflect the strong internal deformation within the Gorda slab^11, 14^. The increased seismicity in northern Cascadia likely indicates strong coupling between the subducting Juan de Fuca plate and the overriding North American plate^15^ and increased dehydration of the oceanic slab^2, 16^. The lack of seismicity in central Cascadia is attributed to an anhydrous or unusually warm segment of the Juan de Fuca slab and the presence of an accreted oceanic terrane within the overriding plate^11, 17, 18^. The small earthquake cluster off central Oregon was associated with the subducted seamounts^12, 13^. However, a comprehensive understanding about the role of the oceanic lithosphere on seismicity, especially prior to subduction, is prevented due to limited constraints for the offshore structures.

**Fig. 2:**
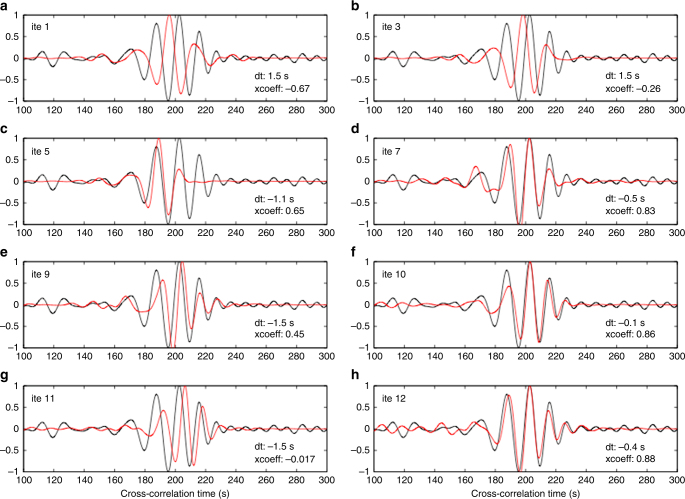
Comparison of observed empirical Green’s functions and synthetic waveforms between the station pair 7D.G10B−TA.I05D for a total of 12 iterations of full-wave simulation and inversion. See station locations in Fig. 1. The waveforms are filtered at 10–25 s periods. The phase delay time and the cross-correlation coefficient between the observed and synthetic data are labeled within each panel. Empirical Green’s functions (black) and synthetic waveforms (red)

Here I present correlations between large-scale variations of the oceanic plates with earthquake distribution patterns both along strike and down dip at the Cascadia subduction system. The three-dimensional seismic structure of the Juan de Fuca and Gorda oceanic plates from formation at the mid-ocean ridge to subduction at the trench is resolved with the use of three-dimensional full-wave simulation and inversion^19–21^. In this study the advanced full-wave ambient noise tomographic method integrates all available seismic stations within the study region from 2004 to 2016 (Fig. 1) into one model using high-quality Rayleigh wave signals at periods of 10–150 s (Supplementary Figure 1). The sensitivities of Rayleigh waves to perturbations of both Vp and Vs are jointly inverted for the velocity model. The velocity model is then improved by iteratively reducing the misfit between the observed and synthetic waveforms. The dense coverage of the seismic stations largely increases the total number of phase delay measurements between the observed and synthetic waveforms used for the model inversion (Supplementary Figure 2), in particular for the offshore structure. A final seismic velocity model is achieved after a total of 12 iterations of wave simulation and inversion, which significantly improves the match between the observed and synthetic waveforms (see Fig.  2 and Supplementary Figures 2 and 3).

## Results

### Seismic feature of the oceanic lithosphere

**Fig. 3:**
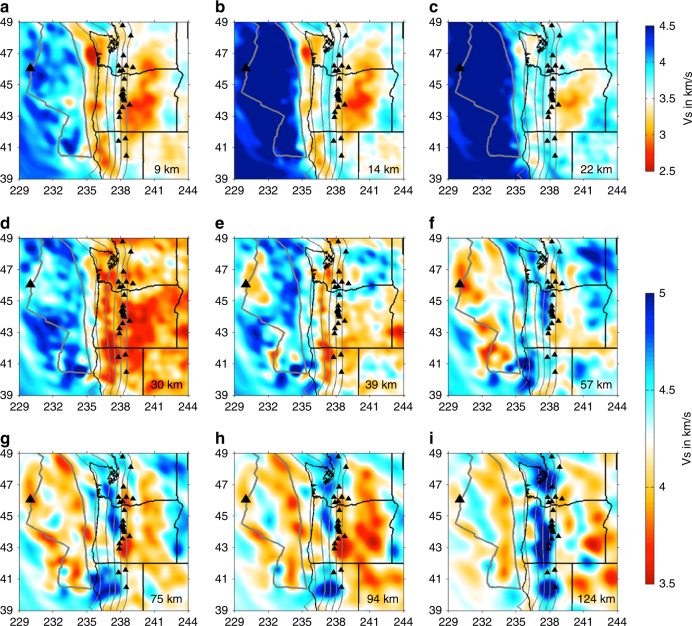
Shear velocity model from the crust down to the upper mantle for the Cascadia subduction system. Note that the maximum resolvable depths are about 75 km for the offshore seismic structure and 150 km for the onshore structure, respectively. Other symbols are the same as in Fig. 1

**Fig. 4:**
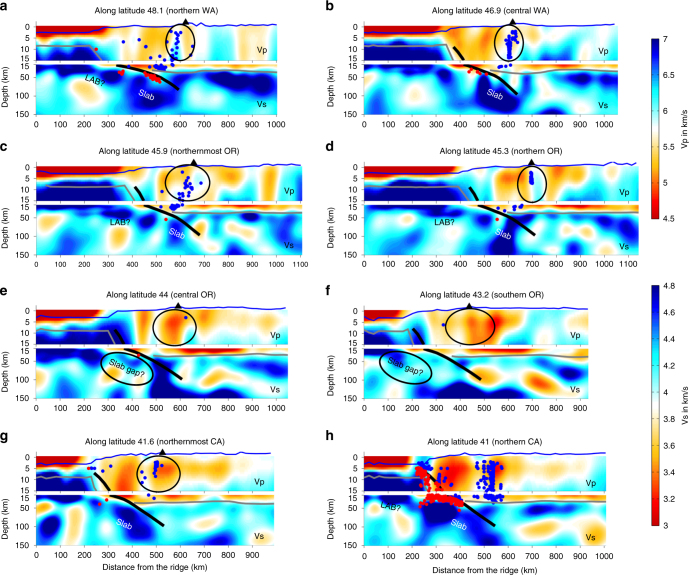
W-E profiles of the seismic tomography model along latitudes, extending from the spreading centers to the Cascade backarc. The upper panels demonstrate the P-wave velocity for the top 15 km depths with ten times vertical exaggeration, and the lower panels are the S-wave velocity at depths of 15–150 km with 1.5 times vertical exaggeration. The blue line represents the bathemetry/topography. The thick black line is the projected plate interface^10^. The gray lines mark the continental Moho^28^ and the oceanic Moho assuming an average oceanic crustal thickness of 6 km. The blue dots are the continental crustal earthquakes and the red dots are the intraslab earthquakes^11^. The black triangle marks the Cascade volcanic front

**Fig. 5:**
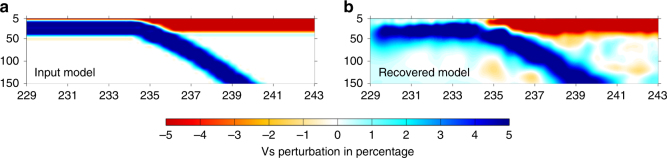
Resolution test of the geometry of the oceanic lithosphere. **a** The input model includes a −5% velocity perturbation for the oceanic and continental crust and a +5% velocity perturbation for the oceanic mantle lithosphere. The thickness of the slab is about 40 km. **b** The recovered shear velocity model

The full-wave ambient noise tomographic imaging demonstrates distinct variation patterns of seismic velocities within the study area (Fig. 3). The low-velocity seismic feature along the Juan de Fuca and Gorda spreading centers has been imaged by many other studies, which is attributed to the presence of partial melting within the upper mantle^19, 22, 23^. The shear-wave velocity amplitude of the oceanic plates varies significantly along strike and down dip (Figs. 1b, 3, 4). West of the trench, the oceanic lithosphere is imaged as a continuous and uniform feature with the thickness less than 40 km. The subducted oceanic plate appears to be a bit thicker and flatter at depths greater than 100 km, which may in fact be an artifact of the tomographic imaging as demonstrated by the model resolution test (Fig. 5). Near the trench, the amplitude of the shear-wave velocity appears to be relatively lower compared to other portions of the slab, indicating a weak segment of the slab. The low shear-velocity feature appears to be strongest beneath central Cascadia (Fig. 4e, f), which extends from the trench down dip to at least 100 km depth for a total length of about 200 km. A low-velocity layer is observed immediately beneath the oceanic lithosphere along the entire subduction zone. Teleseismic P-wave tomography^24^ also imaged a strong low-velocity zone westward of the Juan de Fuca plate extending down to 300 km depth, which was interpreted as an accumulation of buoyant asthenosphere material with low viscosity.

The top of the oceanic lithosphere imaged in this study roughly agrees with the plate interface as defined more accurately by active source seismic data^10, 11^ (thick black lines in Fig. 4), except in central Cascadia where the slab interface is poorly constrained due to the lack of seismic data. Comparison of the slab geometry resolved in this study with other previous tomographic models^25–29^ that used only land stations demonstrates a similar pattern of the Juan de Fuca and Gorda subducting slabs in a large scale (Supplementary Figure 4). For example, the along-strike slab gaps are observed roughly across the Washington-Oregon and Oregon-California state boundaries, which have also been suggested by geochemical studies along the Cascade arc volcanoes^30^. Nevertheless, the amplitude of the shear-wave velocity within the slab varies significantly among these tomographic models, reflecting the impacts of various seismological methods and the data coverage^31–33^. The full-wave ambient noise tomographic model in this study shows the strongest velocity variations compared to other models, which are required by the observed data (see Fig. 2 and Supplementary Figure 3).

### Crustal structure beneath the Cascade volcanic arc

At the shallow crust, where P-wave velocity can be best resolved^19^ (see resolution tests in Supplementary Figures 5 and 6), the seismic structure beneath the Cascade volcanic arc demonstrates remarkable along-strike variations (Fig. 4). In the northern Cascades, a sharp velocity gradient is imaged immediately across the arc front (Fig. 4a–d), while the P-wave velocity appears to be much lower and broader beneath the central and southern Cascades region (Fig. 4e–h). A few factors may contribute to the distinct P-wave velocity variations along the arc and forearc region, such as the accreted oceanic terrane, thermal variations, amounts of subducted sediments, and magmatic melts and fluids, whose contributions cannot be easily distinguished. Magnetotelluric data imaged a sharp resistivity gradient beneath Mount Rainier at depths shallower than 20 km^34^, which was proposed to indicate presence of melt/fluids. Similar as our tomographic imaging, the joint inversion of P- and S-wave travel time data revealed a strong low-high Vp (and Vp/Vs ratio) transition beneath Mount St. Helens within 5–15 km depths^35^, which was inferred as a crustal magma reservoir.

## Discussion

Interestingly, the large-scale variations of the slab geometry imaged in this study correlate well with the distribution patterns of the Cascadia background seismicity (Figs. 1b, 4). In northern and southern Cascadia, where the slab can be clearly imaged from the spreading centers to the arc, both intraslab earthquakes and continental crustal earthquakes are well recorded. In contrast, we observe a weak (or nonexistent) slab near the trench in central Cascadia where seismicity is scarce. The strong spatial linkage suggests that the properties of the incoming oceanic plate play a significant role on the distribution patterns of seismicity both above and below the slab interface. Note that here the definition of the plate interface as well as the earthquake catalog are inferred from previous studies^10, 11^, considering that seismic tomography cannot precisely detect the plate interface due to the smooth horizontal and vertical model resolutions.

Distribution of continental crustal earthquakes at shallow depths also demonstrates very strong correlations with the Vp crustal model (Fig. 4). In western Washington and northernmost Oregon where a sharp velocity gradient is observed beneath the arc front, most crustal earthquakes are located within the fast-velocity zone either directly beneath the arc or immediately west of the arc. There are no crustal earthquakes recorded in central and southern Oregon where the arc is imaged as a relatively larger-scale low-velocity zone. In northwestern California, no strong correlation is observed between the P-wave velocity anomaly and the distribution of the intraslab and continental crustal earthquakes. This is not unexpected as we know that the strong internal deformation of the Gorda plate and the presence of the Mendocino triple junction can significantly complicate the earthquake distribution pattern in northern California^5, 11, 14, 36^.

A few possibilities can significantly weaken the slab near the trench in central Cascadia, resulting in the observed large-scale low-velocity feature, and consequently control the large-scale distribution variations of seismicity at Cascadia. Hydration and alteration of the oceanic mantle lithosphere have been observed near the trench at global subduction zones due to the reactivation and/or formation of bending-related faultings^9, 37–40^. A recent seismic reflection study at Cascadia^41^ showed that bending faults penetrate through the crust and extend into the oceanic mantle off the Oregon margin but are limited within the crust off the Washington margin, suggesting that the oceanic mantle near the Oregon trench is highly hydrated and serpentinized. This supports our tomographic imaging of the observed low-velocity feature near the trench being strongest in central Cascadia. The magnetotelluric data in the Costa Rican subduction zone^40, 42^ observed extremely similar features as the seismic tomographic imaging in Cascadia. That is, the oceanic lithosphere is imaged as a uniform thin layer westward of the trench and is slightly thickened after subduction, with a strong low resistivity near the trench, which was interpreted as hydration and serpentinization of the oceanic mantle lithosphere^40^.

However, it is less likely that hydration and serpentinization of the oceanic mantle lithosphere alone can attribute to the observed low-velocity anomaly near the Cascadia trench considering its scale and depth extent in this study (Fig. 4). It was suggested that the plume−slab interaction could significantly weaken the slab beneath central Oregon but at much greater depths^43^. Mantle upwelling has been previously proposed to explain the observed low seismic velocity beneath the Pacific plate above the mantle transition zone^44^, existence of nearly anhydrous lavas, low seismic velocities and high attenuation at the back-arc spreading center^45, 46^, and low seismic velocities and high conductivities in the Cascade backarc^20, 47^. However, considering the strong three-dimensional mantle flow due to slab rollback, it is less likely that the mantle upwelling and corresponding decompressional melting would accumulate beneath the oceanic lithosphere at shallow depths.

Here I suggest that other mantle dynamic processes need to be considered for the observed seismic features in Cascadia. The low-velocity layer observed beneath the Juan de Fuca and Gorda plate along the entire Cascadia subduction system may represent the oceanic lithosphere−asthenosphere boundary, indicating presence of partial melts, fluids, and/or volatiles as has been proposed by many previous studies^42, 48–51^. However, the nature of the lithosphere−asthenosphere boundary is highly debatable, as well as the sources of melts/fluids/volatiles. Furthermore, the subducting slab appears to bend slightly steeper in central Cascadia^11^ compared to the northern and southern parts, potentially resulting in a higher strain rate and a relatively weaker slab segment near the trench region. Consistently, three-dimensional numerical models for the Alaska subduction system^52^ estimated low viscosity and high strain rate within and below the subducting slab near the trench, showing similar patterns as the tomographically imaged low-velocity feature near the central Cascadia trench region. Another factor that may contribute to the observed low-velocity feature is the axial seamount located in the central Juan de Fuca ridge, where low seismic velocities are observed (Figs. 1b, 3) and presence of partial melting is indicated^19, 22, 23^. The partial melting could migrate away from the ridge and accumulate beneath the trench region, significantly reducing the seismic velocities.

In summary, a highly hydrated oceanic lithosphere near the trench where the plate bends, together with a thin sheared layer of melts/fluids/volatiles underneath, can significantly reduce the shear-wave velocity and lower the strength of the oceanic lithosphere at central Cascadia. The weak segment of the oceanic lithosphere could be partially decoupled from both the underlying asthenosphere and the overriding North American continent, reducing the probabilities of seismicity at central Cascadia. However, how the properties of the incoming oceanic plate actually affect the distribution patterns of background seismicity in Cascadia remains an open question and should be further studied.

## Methods

### Extraction of empirical Green’s functions

The empirical Green’s functions (EGF) are extracted from the ambient noise cross-correlation of vertical-to-vertical components between each station pairs^19–21^. The geophysical data set in the last decade is excellent at Cascadia both offshore and onshore, including the Cascadia Initiative Amphibious Array, the Blanco Transform experiment, the Gorda Deformation Zone experiment, Neptune Canada, the EarthScope Transportable Array, and many flexible array deployments, resulting in a total of more than 800 seismic stations. The dense coverage of seismic stations provides us for the first time an unprecedented opportunity to construct a complete model of the entire subduction system (Fig. 1). In this study, we are able to extract high-quality Rayleigh-wave signals at periods of 10–50 s between the offshore−offshore and offshore−onshore station pairs, and up to 150 s between the onshore−onshore seismic stations (see Supplementary Figure 1). The asymmetry observed between the causal and acausal parts of the EGFs may reflect the non-uniform distribution of the noise sources around the seismic stations, which should have a minor effect on the surface-wave velocities^20^. The phase delay times between the EGFs and synthetic waveforms are measured by cross-correlation at multiple overlapping period bands, ranging from 75–150 s, 50–100 s, 35–75 s, 25–50 s, 15–35 s, to 10–25 s (see example in Fig. 2).

### Finite-difference wave simulation and inversion

The tomographic models we present here are a major upgrade of the previous full-wave tomography^19^, following the same procedures of wave simulation and inversion. The major difference is that we integrate all available offshore and onshore seismic stations from 2004 to 2016 into one model, which largely increases the number of seismic stations from ~200^19^ to >800 in this study. Correspondingly, the total number of phase delay measurements has been increased from 13,500 to over 50,000 used for the model inversion (Supplementary Figure 2). The average cross-correlation coefficient between the observed EGFs and synthetics among all the station pairs has been improved from 0.73^19^ to about 0.9 in this study (see Supplementary Figure 2), and the model resolution has been progressively improved through each iteration (see Supplementary Figure 3). Supplementary Figure 3 also demonstrates that the final velocity model does not depend on the selection of the initial reference model.

### Checkerboard resolution tests

The sensitivity kernels of the Rayleigh waves to shear-wave velocity structure are frequency dependent. That is, the longer the period, the deeper the Vs structure sampled. The checkerboard resolution tests show that the shear-wave velocity model can be well recovered within a depth range of 10–150 km for the continental crust and upper mantle, and of 15–75 km for the oceanic lithosphere. The minimum checkerboard dimensions resolvable in this study increase from 65 to 150 km with depth (see Supplementary Figure 5). In full-wave ambient noise tomography, the Rayleigh waves are primarily sensitive to P-wave velocity at depths shallower than 15 km^19^ (see Supplementary Figure 6). The inclusion of P-wave velocity in inversion provides additional degrees of freedom, which minimize the extent to which P-velocity anomalies at the shallow crust are imaged into greater depths. In this study, P-velocity structures with a horizontal dimension of 50 km or greater can be well imaged at depths of 3–15 km.

### Data availability

All the continuous seismic data are requested from the IRIS Data Management Center (http://ds.iris.edu/ds/nodes/dmc/). The empirical Green’s functions derived from ambient noise, the travel time anomalies between the observed and synthetic waveforms, and the P- and S-wave velocity models are available upon a request to the author.

## Electronic supplementary material

Supplementary Information(PDF 4398 kb)

## References

[1] van Keken PE, Hacker BR, Syracuse EM, Abers GA (2011). Subduction factory: 4. Depth-dependent flux of H2O from subducting slabs worldwide. J. Geophys. Res..

[2] Ji, Y., Yoshioka, S. & Banay, Y. A. Thermal state, slab metamorphism, and interface seismicity in the Cascadia subduction zone based on 3-D modeling. *Geophys*. *Res*. *Lett*. **44**, 10.1002/2017GL074826 (2017).

[3] Atwater BF, Stuiver M, Yamaguchi DK (1991). Radiocarbon test of earthquake magnitude at the Cascadia subduction zone. Nature.

[4] Goldfinger, C. et al. *Turbidite Event History—Methods and Implications for Holocene Paleoseismicity of the Cascadia Subduction Zone*. U.S. Geological Survey Professional Paper 1661-F, 170 (2012).

[5] Wang K, Tréhu AM (2016). Some outstanding issues in the study of great megathrust earthquakes—the Cascadia example. J. Geodyn..

[6] Bilek, S. L. Seismicity along the South American subduction zone: review of large earthquakes, tsunamis, and subduction zone complexity. *Tectonophysics*10.1016/j.tecto.2009.02.037 (2009).

[7] Wagner, L. S., Beck, S. & Zandt, G. Upper mantle structure in the south central Chilean subduction zone (30° to 36°S). *J*. *Geophys*. *Res*. **110**, 10.1029/2004JB003238 (2005).

[8] Wallace LM (2010). Subduction systems revealed: studies of the Hikurangi margin. Eos.

[9] Shillington, D. J. et al. Link between plate fabric, hydration and subduction zone seismicity in Alaska. *Nat. Geosci*. 10.1038/NGEO2586 (2015).

[10] McCrory, P. A., Blair, J. L., Oppenheimer, D. H. & Walter, S. R. Depth to the Juan De Fuca Slab beneath the Cascadia Subduction Margin—a 3-D model for sorting earthquakes. U.S. Geol. Surv. Data Ser., **91**, http://pubs.usgs.gov/ds/91/ (2006).

[11] McCrory PA, Blair JL, Waldhauser F, Oppenheimer. DH (2012). Juan de Fuca slab geometry and its relation to Wadati-Benioff zone seismicity. J. Geophys. Res..

[12] Tréhu AM, Blakely RJ, Williams MC (2012). Subducted seamounts and recentearthquakes beneath the central Cascadia forearc. Geology.

[13] Tréhu AM, Braunmiller J, Davis E (2015). Seismicity of the central Cascadia continental margin near 44.5◦N: a decadal view. Seis. Res. Lett..

[14] Chaytor JD, Goldfinger CR, Dziak P, Fox CG (2004). Active deformation of the Gorda plate: constraining deformation models with new geophysical data. Geology.

[15] McCaffrey R (2009). Time-dependent inversion of three-component continuous GPS for steady and transient sources in northern Cascadia. Geophys. Res. Lett..

[16] Preston LA, Creager KC, Crosson RS, Brocher TM, Tréhu AM (2003). Intraslab earthquakes: dehydrating the Cascadia slab. Science.

[17] Bostock MG, Hyndman RD, Rondenay S, Peacock SM (2002). An inverted continental Moho and serpentinization of the forearc mantle. Nature.

[18] Peacock, S. M., Wang, K. & McMahon, A. M. *Thermal Structure and Metamorphism of Subducting Oceanic Crust—Insight into Cascadia Intraslab Earthquakes*. U.S. Geol. Survey Open-File Report 02-328, 123–126 (2002).

[19] Gao, H. Seismic velocity structure of the Juan de Fuca and Gorda plates revealed by a joint inversion of ambient noise and regional earthquakes. *Geophys*. *Res*. *Lett*. **43**, 10.1002/2016GL069381 (2016).

[20] Gao H, Shen Y (2014). Upper mantle structure of the Cascades from full-wave ambient noise tomography: evidence for 3D mantle upwelling in the back-arc. Earth Planet. Sci. Lett..

[21] Gao, H. & Shen, Y. A preliminary full-wave ambient noise tomography model spanning from the Juan de Fuca and Gorda spreading centers to the Cascadia volcanic arc. *Seismological Res. Lett.* **86**, 10.1785/0220150103 (2015).

[22] Bell S, Ruan Y, Forsyth DW (2016). Ridge asymmetry and deep aqueous alteration at the trench observed from Rayleigh wave tomography of the Juan de Fuca plate. J. Geophys. Res. Solid Earth.

[23] Byrnes, J. S., Toomey, D. R., Hooft, E. E. E., Nabelek J. & Braunmiller, J. Mantle dynamics beneath the discrete and diffuse plate boundaries of the Juan de Fuca plate: results from Cascadia Initiative body wave tomography. *Geochem*. *Geophys*. *Geosyst*. **18**, 10.1002/2017GC006980 (2017).

[24] Hawley WB, Allen RM, Richards MA (2016). Tomography reveals buoyant asthenosphere accumulating beneath the Juan de Fuca plate. Science.

[25] Porritt RW, Allen RM, Pollitz FF (2014). Seismic imaging east of the Rocky Mountains with USArray. Earth Planet. Sci. Lett..

[26] Porter R, Liu Y, Holt WE (2016). Lithospheric records of orogeny within the continental U.S. Geophys. Res. Lett..

[27] Schmandt B, Lin FC (2014). P and S wave tomography of the mantle beneath the United States. Geophys. Res. Lett..

[28] Schmandt B, Lin FC, Karlstrom KE (2015). Distinct crustal isostasy trends east and west of the Rocky Mountain Front. Geophys. Res. Lett..

[29] Shen W, Ritzwoller MH (2016). Crustal and uppermost mantle structure beneath the United States. J. Geophys. Res. Solid Earth.

[30] Mullen EK, Weis D, Marsh NB, Martindale M (2017). Primitive arc magma diversity: new geochemical insights in the Cascade Arc. Chem. Geol..

[31] Becker TW (2012). On recent seismic tomography for the western United States. Geochem. Geophys. Geosyst..

[32] Gao H, Shen Y (2012). Validation of shear-wave velocity models of the Pacific Northwest. Bull. Seism. Soc. Am..

[33] Gao H, Shen Y (2015). Validation of recent shear wave velocity models in the United States with full-wave simulation. J. Geophys. Res. Solid Earth.

[34] McGary, R. S., Evans, R. L., Wannamaker, P. E., Elsenbeck, J. & Rondenay, S. Pathway from subducting slab to surface for melt and fluids beneath Mount Rainier. *Nature*10.1038/nature13493 (2014) 10.1038/nature13493 25030172

[35] Kiser E (2016). Magma reservoirs from the upper crust to the Moho inferred from high-reoslution Vp and Vs models beneath Mount St. Helens, Washington State, USA. Geology.

[36] Chen X, McGuire JJ (2016). Measuring earthquake source parameters in the Mendocino triple junction region using a dense OBS array: implications for fault strength variations. Earth Planet. Sci. Lett..

[37] Contreras-Reyes E (2011). Deep seismic structure of the Tonga subduction zone: implications for mantle hydration, tectonic erosion, and arc magmatism. J. Geophys. Res. Solid Earth.

[38] Ranero CR, Sallarès V (2004). Geophysical evidence for alteration of the crust and mantle of the Nazca Plate during bending at the north Chile trench. Geology.

[39] van Avendonk HJA, Holbrook WS, Lizarralde D, Denyer P (2011). Structure and serpentinization of the subducting Cocos plate offshore Nicaragua and Costa Rica. Geochem. Geophys. Geosys..

[40] Worzewski T, Jegen M, Kopp H, Brasse H, Castillo W (2011). Magnetotelluric image of the fluid cycle in the Costa Rican subduction zone. Nat. Geosci..

[41] Han S (2016). Seismic reflection imaging of the Juan de Fuca plate from ridge to trench: new constraints on the distribution of faulting and evolution of the crust prior to subduction. J. Geophys. Res. Solid Earth.

[42] Naif, S., Key, K., Constable, S. & Evans, R. L. Melt-rich channel observed at the lithosphere-asthenosphere boundary. *Nature*10.1038/nature11939 (2013). 10.1038/nature11939 23518564

[43] Cheng, C., Bodin, T., Tauzin, B. & Allen, R. M. Cascadia subduction slab heterogeneity revealed by three dimensional receiver function Kirchhoff migration. *Geophys*. *Res*. *Lett*. **44**, 10.1002/2016GL072142 (2017).

[44] Ismail-Zadeh A, Honda S, Tsepelev I (2013). Linking mantle upwelling with the lithosphere descent and the Japan Sea evolution: a hypothesis. Nature.

[45] Conder, J. A., Wiens, D. A. & Morris, J. On the decompression melting structure at volcanic arcs and back-arc spreading centers. *Geophys*. *Res*. *Lett*. **29**, 10.1029/2002GL015390 (2002).

[46] Wiens DA, Conder JA, Faul UH (2008). The seismic structure and dynamics of the mantle wedge. Annu. Rev. Earth Planet. Sci..

[47] Meqbel NM, Egbert GD, Wannamaker PE, Kelbert A (2014). Deep electrical resistivity structure of the northwestern U.S. derived from 3-D inversion of USArray magnetotelluric data. Earth Planet. Sci. Lett..

[48] Kawakatsu H (2009). Seismic evidence for sharp lithosphere-asthenosphere boundaries of oceanic plates. Science.

[49] Sakamaki, T. et al. Ponded melt at the boundary between the lithosphere and asthenosphere. *Nat. Geosci*. 10.1038/NGEO1982 (2013).

[50] Schmerr N (2012). The Gutenberg discontinuity: melt at the lithosphere-asthenosphere boundary. Science.

[51] Stern TA (2015). A seismic reflection image for the base of a tectonic plate. Nature.

[52] Jadamec MA, Billen MI (2010). Reconciling surface plate motions with rapid three-dimensional mantle flow around a slab edge. Nature.

