# Supplementary material for: Three-dimensional variations of the slab geometry correlate with earthquake distributions at the Cascadia subduction system
Source: Nat Commun. 2018 Mar 23;9:1204. doi: 10.1038/s41467-018-03655-5 (PMC5865183; doi:10.1038/s41467-018-03655-5)
Supplement: Supplementary file 1 — Supplementary Information(PDF 4398 kb) [file 41467_2018_3655_MOESM1_ESM.pdf]

Three-dimensional variations of the slab geometry correlate with earthquake distributions at the Cascadia subduction system

Haiying Gao*

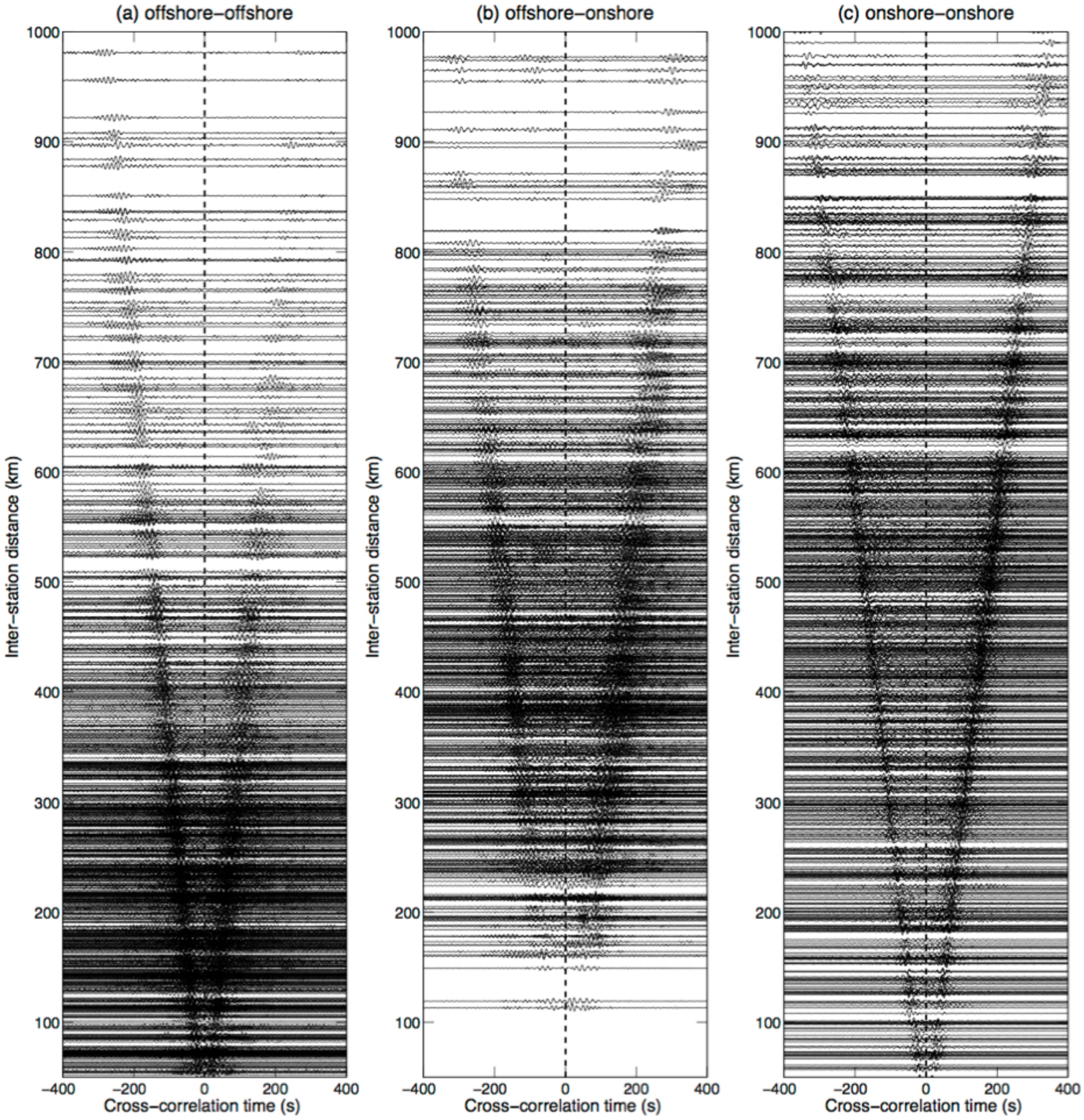

Supplementary Figure 1. Examples of empirical Green's functions derived from ambient noise cross-correlation of vertical-to-vertical components. (a), (b), and (c) show

the empirical Green's functions between offshore-offshore, offshore-onshore, and onshore-onshore seismic station pairs, filtered at periods of 10-50 s, 10-50 s, and 10-150 s, respectively.

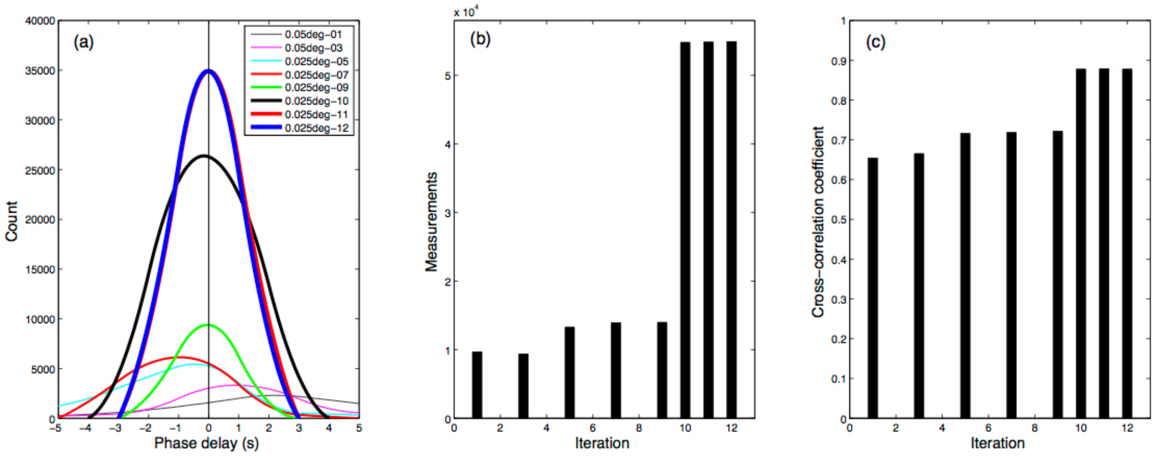

Supplementary Figure 2. (a) Histogram of the phase delay measured between the observed empirical Green's functions and the synthetic waveforms for 12 iterations. (b) Number of phase delay measurements used for tomographic inversion for each iteration. The sharp increase in the measurement of phase delays from iteration 9 to iteration 10 reflects the increase of the station numbers from ~200 to over 800. (c) Average cross-correlation coefficient between the observed and synthetic waveforms from all the station pairs at all the period bands used in wave simulation and inversion. Both the number of measurements and the average cross-correlation coefficient have been progressively increased through the 12 iterations, reflecting the improvement of the model resolution.

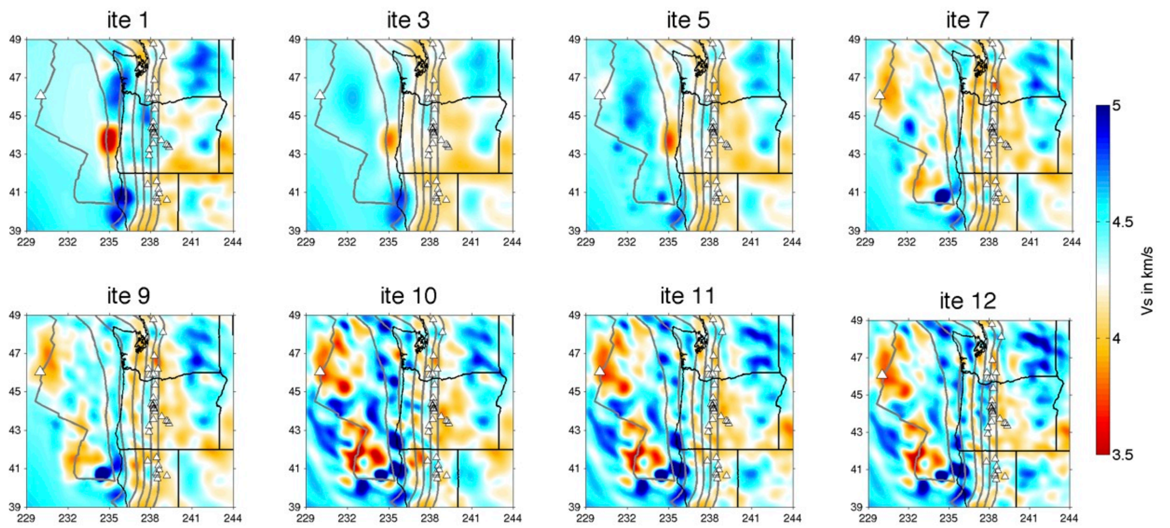

Supplementary Figure 3. Comparison of shear-wave velocity model at 50 km depth for the 12 iterations of wave simulation and inversion. Other symbols are the same as Fig. 1.

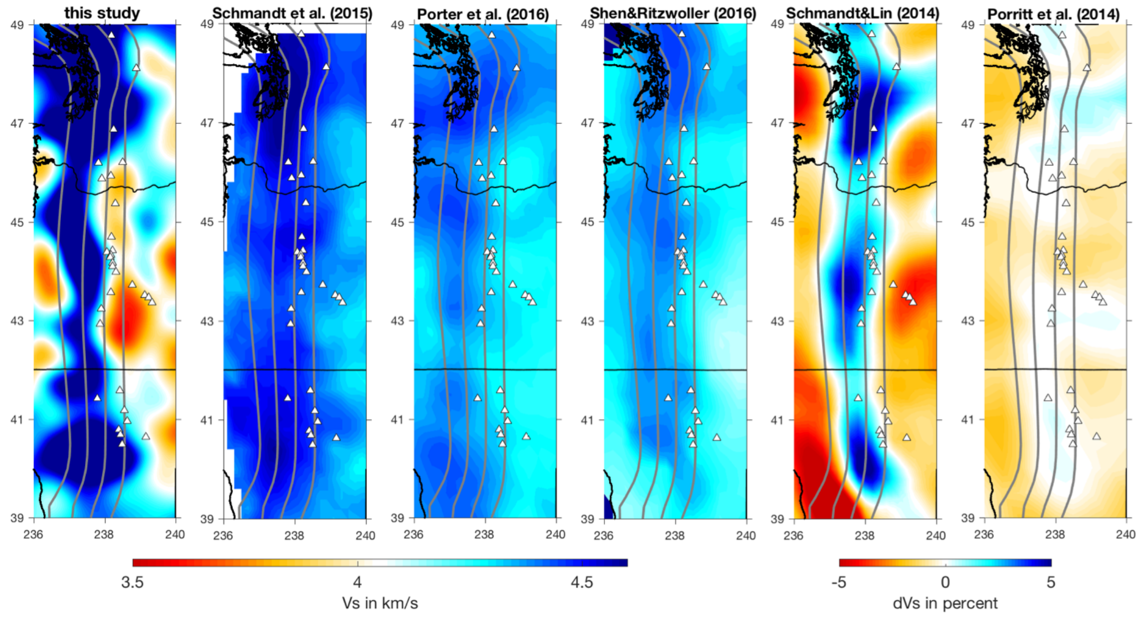

Supplementary Figure 4. Comparison of the imaged subducting slab at 100 km depth along the Cascadia subduction zone from multiple studies[1,2,3,4,5]. The gray contours are the depth contours of the Juan de Fuca plate interface at 40, 60, 80, and 100 km[6]. The white triangles mark the Cascade arc volcanoes.

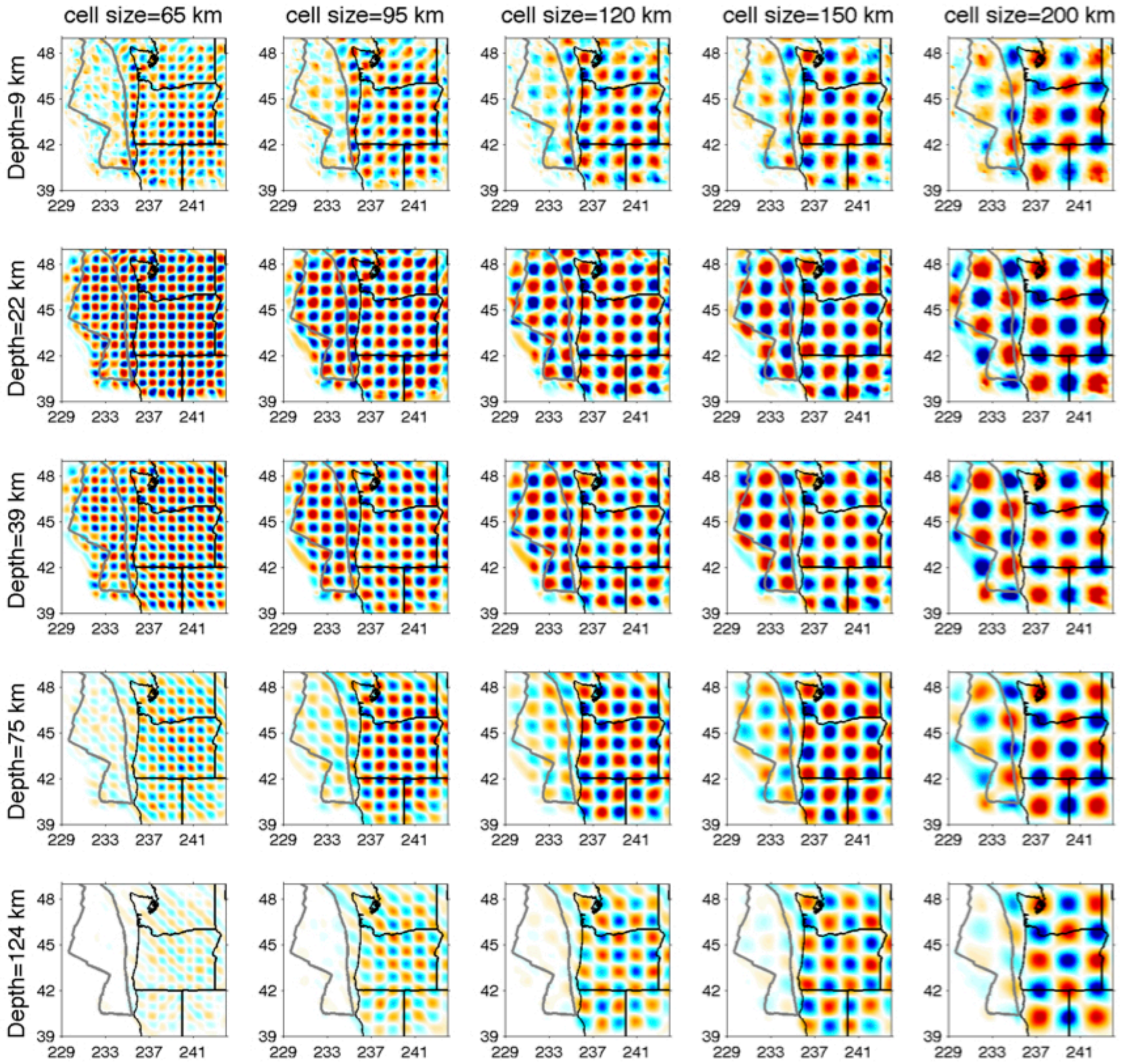

Supplementary Figure 5. Checkerboard resolution tests for shear-wave velocity model at depths of 9-124 km. The horizontal dimension of the checkerboard cells for the input model varies from 65 km to 200 km, with a ±10% velocity perturbation. The resolvable horizontal scale increases with depth. At depths greater than 75 km, the onshore seismic structure can be much better resolved than offshore.

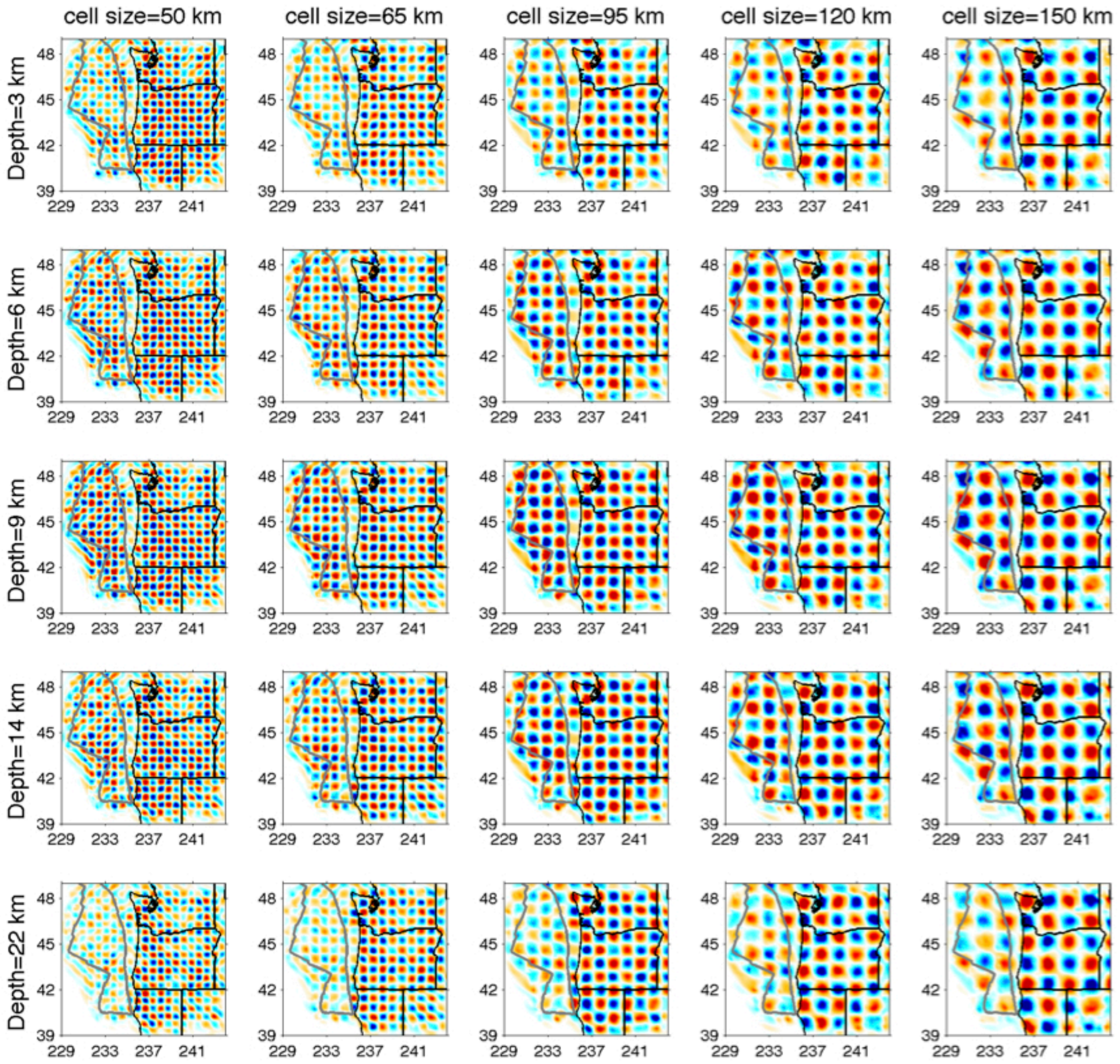

Supplementary Figure 6. Checkerboard resolution tests for P-wave velocity model at depths of 3-22 km. The horizontal dimension of the checkerboard cells for the input model varies from 50 km to 150 km, with a ±10% velocity perturbation. Both the offshore and onshore seismic structures with a horizontal scale of 50 km or greater can be well recovered at depths shallower than 15 km.

Supplementary References

1. Schmandt, B., F. C. Lin, and K. E. Karlstrom (2015), Distinct crustal isostasy trends east and west of the Rocky Mountain Front, Geophys. Res. Lett., 42, 10,290-10,298, doi:10.1002/2015GL066593.
2. Porter, R., Y. Liu, and W. E. Holt (2016), Lithospheric records of orogeny within the continental U.S., Geophys. Res. Lett., 43, 144-153, doi:10.1002/2015GL066950.
3. Shen, W., and M. H. Ritzwoller (2016), Crustal and uppermost mantle structure beneath the United States, J. geophys. Res. Solid Earth, 121, 4306-4342, doi:10.1002/2016JB012887.
4. Schmandt, B., and F.-C. Lin (2014), P and S wave tomography of the mantle beneath the United States, Geophys. Res. Lett., 41, 6342-6349, doi:10.1002/2014GL061231.
5. Porritt, R. W., R.M. Allen, and F. F. Pollitz (2014), Seismic imaging east of the Rocky Mountains with USArray, Earth Planet. Sci. Lett., 402, 16-25, doi:10.1016/j.epsl.2013.10.034.
6. McCrory, P. A., J. L. Blair, D. H. Oppenheimer, and S. R. Walter (2006), Depth to the Juan De Fuca Slab beneath the Cascadia Subduction Margin - A 3-D model for sorting earthquakes, U.S. Geol. Surv. Data Ser., 91, http://pubs.usgs.gov/ds/91/.
